# How is visual salience computed in the brain? Insights from behaviour, neurobiology and modelling

**DOI:** 10.1098/rstb.2016.0113

**Published:** 2017-02-19

**Authors:** Richard Veale, Ziad M. Hafed, Masatoshi Yoshida

**Affiliations:** 1 Department of System Neuroscience, National Institute for Physiological Sciences, Okazaki, Japan; 2 Physiology of Active Vision Laboratory, Werner Reichardt Centre for Integrative Neuroscience, University of Tuebingen, Tuebingen, Germany; 3 School of Life Science, The Graduate University for Advanced Studies, Hayama, Japan

**Keywords:** overt attention, saliency map, superior colliculus, lateral inhibition, microsaccades, spiking neuron network

## Abstract

Inherent in visual scene analysis is a bottleneck associated with the need to sequentially sample locations with foveating eye movements. The concept of a ‘saliency map’ topographically encoding stimulus conspicuity over the visual scene has proven to be an efficient predictor of eye movements. Our work reviews insights into the neurobiological implementation of visual salience computation. We start by summarizing the role that different visual brain areas play in salience computation, whether at the level of feature analysis for bottom-up salience or at the level of goal-directed priority maps for output behaviour. We then delve into how a subcortical structure, the superior colliculus (SC), participates in salience computation. The SC represents a visual saliency map via a centre-surround inhibition mechanism in the superficial layers, which feeds into priority selection mechanisms in the deeper layers, thereby affecting saccadic and microsaccadic eye movements. Lateral interactions in the local SC circuit are particularly important for controlling active populations of neurons. This, in turn, might help explain long-range effects, such as those of peripheral cues on tiny microsaccades. Finally, we show how a combination of *in vitro* neurophysiology and large-scale computational modelling is able to clarify how salience computation is implemented in the local circuit of the SC.

This article is part of the themed issue ‘Auditory and visual scene analysis’.

## 1. Visual scene analysis in the brain

The brain responds to the visual world via a collection of parallel neural pathways beginning in the retina. Some of these pathways perform selective modulation of the visual signal, highlighting features and locations that contain relevant information. Because we can only look at one location at a time, such selectivity allows us to sequentially sample the visual world by moving our eyes, head and body. We refer to this redirection of sensory apparati as ‘overt attention’. This review lays out the current state of neurobiological evidence for overt attention. In other words, how does the brain select the next place to look? Evidence is converging to support the hypothesis that there exist multiple ‘maps’ in the brain that participate in computing the next place to look. Within each map, the conspicuity of all points in the visual scene is encoded in parallel. The next target of attention is then selected via a process involving competition within each map and merging of maps.

Two types of map have been proposed. One type is the ‘saliency map’ [1], which computes visually conspicuous points based on low-level visual features such as brightness, colour, oriented edges and motion. The other map type is known as the goal-directed ‘priority map’ [2–4]. The priority map integrates information from the bottom-up saliency map with task- and goal-relevant information. Neither the saliency map nor the priority map exclusively encodes the target that has been selected to look at next. Rather, the maps code the graded salience or priority values for each location in the visual field. Even though the target of the next saccade may not yet be selected, each map contains information about the probability of a visual location being next foveated. It is important to clarify this terminology, because different authors use the word ‘attention’ to refer to different physiological, behavioural or cognitive phenomena. Here, we take care to differentiate between ‘graded attention’ representations (pre-selection) versus ‘attentional target’ representations (post-selection). We focus on how overt attention (i.e. the point in visual space that is being fixated) is influenced by both pre- and post-selection maps.

Using experiments that dissociate the contributions of low-level saliency maps from goal-directed priority maps, a picture has begun to emerge for how the brain is able to use a combination of bottom-up and top-down mechanisms to efficiently select the next attentional target. This review addresses our understanding of the neural circuits that underlie the bottom-up saliency map, and specifically how these circuits contribute to saccadic eye movements, which represent the fastest way to redirect overt attention. Besides clarifying computational principles and underlying neurophysiological mechanisms, our review complements clinical perspectives in the study of visual (and auditory) salience. For example, it is known that individuals with autism spectrum disorders (ASD) perform differently in both visual and auditory scene analysis tasks than non-ASD individuals [5]. Thus, understanding the mechanisms responsible for overt attention shifts can aid in differential diagnosis and possibly even therapy. Although this review focuses on visual stimuli, sounds also commonly draw overt attention shifts. Similar to how ‘colour’ is used to compute salience in the visual modality, Southwell *et al.* [6] have found that one salient property of auditory stimuli is ‘predictable repetition over time’ . For a broader background, this issue also includes comprehensive review comparing models of auditory and visual salience [7].

Our review proceeds as follows. First, we present a short overview of the bottom-up saliency map model, so that it can be clearly dissociated from goal-directed priority, and from visual feature analysis. Second, we overview attention-related visual pathways of the brain, focusing on physiological and behavioural evidence for saliency map-like or priority map-like responses in these pathways. We conclude that although priority-map-like and saliency-map-like responses can be observed in various areas, one brain region in particular—the superior colliculus (SC)—mechanistically implements the saliency map computational model by virtue of its local circuits and unique pattern of inputs and outputs. Third, in the light of this, we zoom in to focus on the SC. The SC is a midbrain structure that has emerged as a strong candidate for being the final gatekeeper between saliency/priority maps and overt behaviour. In order to support this hypothesis, we review SC anatomy and physiology in detail, complemented with recent in-depth computational models fit to empirical data.

## 2. What is a saliency map?

**Figure 1.**
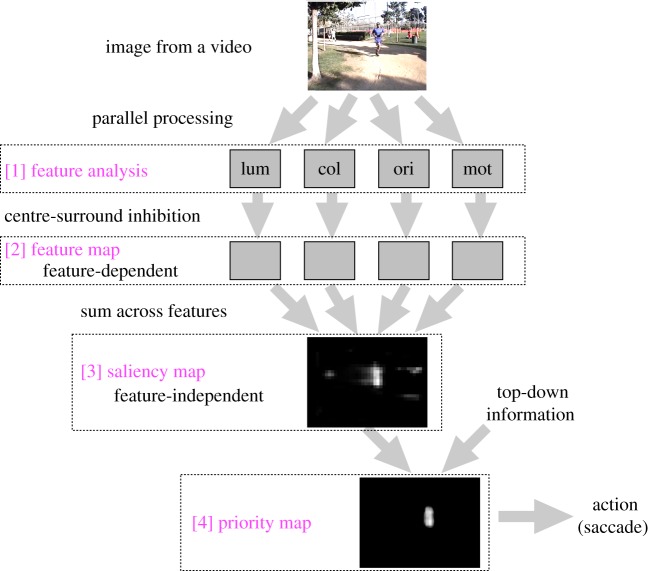
Overview of the major steps of the Itti salience model. Visual information is analysed in parallel at feature analysis [1] and is used to detect conspicuous locations at feature maps [2]. Then, the feature maps are combined to make a feature-agnostic saliency map [3]. Then, it is combined with top-down information to make a priority map [4]. Lum stands for luminance feature, col for colour feature, ori for orientation feature and mot for motion feature. (Online version in colour.)

A salience computational model describes how low-level exogenous visual features such as colour, orientation, luminance and motion are combined into a single global map representing the relative ‘salience’ of each point on the map. The saliency map is a two-dimensional map, with the amplitude at a given point representing how perceptually conspicuous the corresponding region is in visual space, regardless of what caused it to be conspicuous. In other words, the saliency map is feature-agnostic—a highly salient point could equally have been caused by a yellow dot on a blue background as by a non-moving region against a moving background. The saliency map concept was originally proposed by Koch & Ullman [1] and was later implemented by Itti *et al.* [8,9]. We refer to this implementation as the Itti salience model. Figure 1 overviews the major pieces of the saliency map computational model. In short: (i) feature maps representing basic visual features such as colour, orientation, luminance and motion (computed from image sequences) compete within themselves to determine which locations on the map are most ‘different’ from their surroundings at many spatial scales; (ii) feature maps are normalized and then combined into a feature-agnostic ‘saliency map’. The saliency map is then used to determine the most likely target for attention. Variations of this basic saliency map model have been extensively applied to predicting human eye movements during free-viewing of natural and complex scenes [10–12]. Hereafter, we use terminology primarily following the Itti salience model [8,10,13], such as ‘feature map’, ‘saliency map’ and ‘priority map’.

Despite success in predicting eye movements, it is not clear what the saliency map represents from a neural standpoint. In a recent review, Zelinsky & Bisley [14] dissected the theoretical properties needed to differentiate between salience and priority based on the behavioural task. Importantly, they also distinguished between whether a brain area is part of the local computation of salience or priority and whether it receives a computed result as input (‘inheritance’). There have also been other previous reviews about visual attention, which have primarily focused on the computational problems solved by a salience-driven system [13]. Based on these reviews, a prevalent state of the field is that biologically plausible models remain to be developed [13]. However, drawing parallels between computational models and neural activity is a delicate endeavour. Predicting behaviour by the Itti salience model only implies computational similarity between the model and its biological implementation [15]. Furthermore, even if we give the saliency map model the benefit of the doubt, then the same input–output mapping could potentially be accomplished via multiple algorithms. For example, in a digital computer, numbers can be represented in binary or hexadecimal format, and sorting a list of numbers could be accomplished by any number of algorithms, all of which produce the same output. In this review, we are explicitly interested in finding evidence of saliency map model computation in the brain. We look for evidence of computational equivalence (to show that the Itti salience model is the correct computational model) and then algorithmic equivalence (to show that, furthermore, representation of intermediate steps is basically the same set of two-dimensional amplitude maps predicted by the model). We then attempt to understand how the algorithmic equivalence may be realized by the specific implementation of local computations in the spiking neural substrate of the brain.

In §3, we review the corpus of excellent research regarding the neural correlates of salience computation. Over the years, authors have had different interpretations of what it means to be a neural correlate of salience computation, making it difficult to construct a consistent story at any level of description. With this in mind, there is converging evidence that certain brain regions exhibit neural activity that is both retinotopically organized and proportional to the activity predicted by different steps in the saliency map model. In §4, we provide stronger evidence that the brain implements the saliency map model, using recent research from the well-understood subcortical route. We also overview recent results showing how small saccadic eye movements made during fixation (microsaccades) can give insights into local interactions within the SC, and thus constrain the salience model implementation in the brain. Finally, we use biological models fit to physiological data suggesting how salience is *implemented* in local circuits.

## 3. Visual pathways for salience

**Figure 2.**
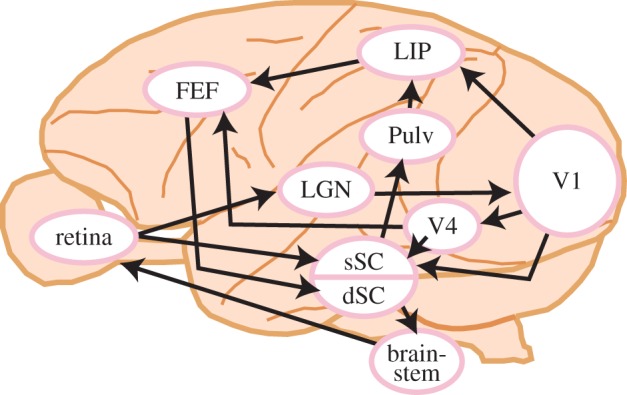
Information flow from retinal input to eye movement output in the macaque brain. Visual signals from the retina to the cerebral cortex are mediated through V1 (cortical pathway) and the SC (subcortical pathway). The cortical pathways eventually project back to the SC, which is connected to the output oculomotor nuclei. There is also a shortcut from the sSC to the dSC. Note that only the pathways dealt with in this review in detail are displayed. For example, the sSC receives input not only from V1 [16] and V4 [17], but also from extrastriate areas V2, MT and TEO [17–19]. LGN, lateral geniculate nucleus; V1, primary visual cortex; LIP, lateral intraparietal area; FEF, frontal eye field; Pulv, pulvinar; sSC, superficial layers of the superior colliculus; dSC, deeper layers of the superior colliculus. (Online version in colour.)

Several parallel pathways control visually guided overt attention shifts (saccades). These pathways all begin in the retina and terminate at the extraocular muscles. Figure 2 shows the major pathways and brain regions addressed in this review. At the sensory side, visual information usually enters the brain via the primary visual cortex (V1), through relays in the lateral geniculate nucleus (LGN) of the dorsal thalamus. At the motor side, eye movements are usually evoked through bursting activity in the deeper layers of the SC, which propagates to eye movement control centres in the brainstem.

Anatomically, V1 sends axons to higher visual areas, such as V4 and the lateral intraparietal area (LIP) [20], as well as to the superficial layers of the SC (sSC), located in the midbrain [21]. There are also parallel projections via other cortical areas to the frontal eye fields (FEF) [20] and then to the deeper layers of the SC (dSC). Such SC projections are both direct [22] and through a disinhibitory pathway via the basal ganglia known to be involved in voluntary gaze shifts [23]. There is also a parallel subcortical route directly from the retina to the sSC, which has been the subject of less attention [24], as well as several other parallel routes to cortex via pulvinar [25]. Neurons in the sSC receive input not only from V1 [16], but also from extrastriate areas V2, V4, MT and TEO [17–19].

To understand how salience is represented in the brain, we must define salience from a neural perspective. For a brain area to represent salience, neurons in the area should exhibit two properties: (i) be selective to salience rather than to visual features *per se*; (ii) have receptive fields (RFs) organized into a two-dimensional topographical map of visual space. Based on this definition, previous papers suggest that there may be no single saliency map in the brain, which represents purely bottom-up visual information with invariance to low-level visual features (e.g. luminance, colour, orientation and motion). Rather, maps are distributed in various areas, with map properties being similar across neighbouring areas [26]; this is reasonable given the bidirectional nature of connectivity between areas. Additionally, experimental data from converging sources (detailed in §§3*a*–*c*) have argued for a role of the areas in figure 2 in one or more of the following functions: (i) feature analysis, which is part of raw visual feature computation rather than salience computation; (ii) feature map representation, in which bottom-up salience computation based on raw visual features is computed; (iii) saliency map representation, using feature-agnostic bottom-up salience computation; and (iv) priority map formation in which behavioural relevance is integrated.

**Figure 3.**
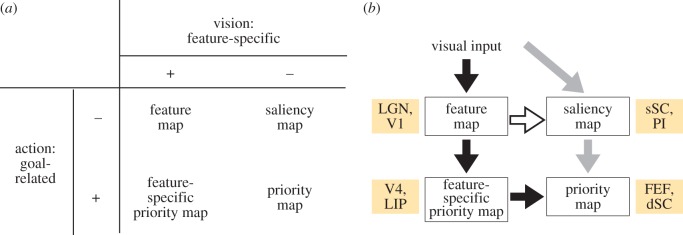
(*a*) Four logically possible maps for salience computation. Columns indicate differences in a visual factor (i.e. whether the map is specialized for certain visual features or not), and rows indicate differences in an action-related factor (i.e. whether the map contains information about behavioural goals or not). Note that in our view, even saccades during free-viewing have a goal in a minimal sense (e.g. SC motor-related neurons would burst for individual saccades during free-viewing). Thus, even during free-viewing tasks that are driven by purely bottom-up signals, the individual saccades during such viewing may still be executed via a priority map as in (*b*). (*b*) Cortical (black arrows) and subcortical (grey arrows) routes for salience computation proposed in this review. The arrow from V1 to sSC is in white to indicate that it is not clear what kind of information is transferred from V1 to sSC. See §3b for detail. PI, inferior pulvinar. (Online version in colour.)

From a neurobiological perspective, we also argue that there are additional constraints on how salience is implemented. Specifically, visual saliency maps may be further classified as exhibiting different emphasis on either vision or action (figure 3*a*). Thus, logically, there are four possible maps, classified into two-by-two components. Each column in figure 3*a* indicates whether the map is specialized for certain visual features or not, and each row indicates whether the map contains information about behavioural goals or not. Thus, the labels ‘vision’ and ‘action’ in figure 3 highlight the specialization within a given map, from a computational modelling sense. In this scheme, feature maps, saliency maps and priority maps can all be classified into one of the four matrix positions (figure 3*a*). The major view of how visually guided overt attention works has been as follows: an implementation of the Itti salience model somewhere in the brain processes visual feature maps into a feature-agnostic saliency map, and then this bottom-up salience information feeds into a priority map where it is integrated with top-down information. However, there is one remaining, logically possible map, having both feature specificity and goal information simultaneously. We call this map a ‘feature-specific priority map’. In the following sections, we classify each relevant brain region as *computationally equivalent* (i.e. having similar output) to one of the four categories of figure 3*a* using data from available human and monkey studies.

### (a) Cortical pathways

#### (i) Lateral geniculate nucleus, visual cortex

Neurons in the retina, LGN and V1 are tuned to visual features such as luminance contrast, colour [27,28] and orientation [29]. Furthermore, intrinsic interactions within V1 and LGN cause neurons to spatially suppress adjacent neurons of the same feature tuning [30–32]. This local suppression means that neural activity in V1 and LGN represents local feature differences, rather than raw visual features. A V1 neuron tuned to respond to red colours will respond to a red dot in its RF less vigorously if the dot is surrounded by other red dots than if it is surrounded by green dots. Thus, V1 computes salience of an odd-ball stimulus, albeit in a *feature-specific* manner.

Although viable proposals exist suggesting that V1 may compute a *feature-agnostic* saliency map [33], these proposals are weakened by the lack of neural data to support them. Recently, one intriguing study used a visual search paradigm with various levels of conjunctive features to demonstrate salience-based behavioural effects [34]. Because V1 neurons are never tuned to conjunctions of visual features, the authors argued that V1 could mediate behavioural effects by implementing a feature-agnostic saliency map. However, behavioural results do not necessitate that the saliency map be implemented in V1. Furthermore, recent results have directly contradicted the hypothesis by providing evidence that blood-oxygen-level dependent (BOLD) signals in V1 do not correlate with salience, but rather with luminance contrast [35]. This is significant, because contrast correlates strongly with salience unless care is taken to separate them. Chen *et al.* [36] responded by measuring BOLD activity while subjects performed a visual discrimination task involving an unrelated natural image presented briefly. The natural images were carefully selected to have single isolated regions of either high or low salience. Chen *et al.* [36] found that V1 BOLD signals were higher for high salience images than low salience images, whereas this was not the case in LGN, V2, V4, LOC or IPS. In contrast, White *et al.* used electrophysiological recordings in macaques to show that SC neurons downstream of V1 certainly do encode salience, whereas neurons in V1 do not (an abstract at Vision Science Society meeting 2014 [37] and [38]). These seemingly contradictory results should make one pause, but White *et al.*’s results are supported by the lesion studies of Yoshida and co-workers, which are presented next.

Further evidence that V1 contributes to salience computation by implementing feature maps is provided by the work of Yoshida *et al.* [39]. These authors used a computational saliency map model [8,40] to predict eye movement patterns of macaques with V1 lesions during free-viewing. Using regression techniques to weigh the contributions of each feature map to the final saliency map, they demonstrated that V1 removal abolished the contribution of orientation features, whereas other feature types (such as luminance, colour and movement) were mostly unaffected. In other words, the monkeys still made eye movements as predicted by the saliency map model even after V1 lesions, but the feature types unambiguously computed in V1 no longer contributed to the looking behaviour of the animals. This work provides us strong support for the hypothesis that pathways beyond V1 are able to compute salience. We will argue later that the most likely candidate in such pathways is the sSC based on its particular pattern of intrinsic and extrinsic connectivity. Overall, the combination of electrophysiological findings supplemented by lesion studies of Yoshida *et al.* [39] strongly supports the idea of V1 being classified into the feature map category of figure 3*a*.

#### (ii) V4

Visual area V4 is an extrastriate cortical area. V4 neurons are tuned to more abstract properties than V1/V2 (e.g. colours or specific shapes) and have RFs of up to a few degrees wide. V4 receives direct input from the early visual cortices V1/V2, and it is strongly modulated by frontal cortical regions (specifically the FEF [41]). This modulation is related to a stimulus being the target of a task [42]. In fMRI experiments, V4 exhibits graded responses to orientation pop-out, which is suggestive of salience computation [43]. Mazer & Gallant [44] examined the role of V4 in selective attention during a free-viewing visual search task. They analysed whether V4 activity predicted the direction of the next eye movement, or whether it was highly correlated with contrast or brightness. They found that activity was related to where the eye would move, but it was locked to stimulus onset. Thus, V4 has a perceptually mediated (bottom-up) guiding role in selecting the next attended target. However, they also found strong top-down modulation. Ogawa & Komatsu [45] found the same pattern of early singleton pop-out. However, the early singleton pop-out response was always followed by modulation that highlighted the behaviourally relevant stimulus. In summary, V4 integrates bottom-up information from the cortical route with goal-related priority information, and communicates this information to downstream brain regions that select the attention target. Because V4's responses are modulated by specific features of a search target, we classify V4 into the feature-specific priority map component of figure 3*a*.

#### (iii) Lateral intraparietal area

The LIP area (IPS in humans) is a parietal region in the dorsal processing stream with subregions whose BOLD signal has been reported to correlate with computational salience [46]. Bogler and co-workers specifically investigated whether the BOLD signal measured from various brain regions correlated linearly with salience, or whether the signal correlated with the most salient point only. The former would suggest a graded saliency map representation, whereas the latter would suggest a winner-take-all representation. They found that the anterior IPS and FEF represented only the final target. In contrast, the visual cortex and the posterior IPS correlated linearly with the salience level of the corresponding visual region. These studies follow those of Gottlieb *et al.* [47], who investigated whether LIP neurons represented the target of the next saccade in a visual search task. The responses to a stimulus brought into neurons' RFs were much stronger when the target was relevant to the task. However, this effect was also observed when stimuli suddenly appeared, confounding bottom-up and top-down salience. Buschman & Miller [48] recorded from the LIP and FEF simultaneously. They found that LIP neurons responded earlier to the bottom-up aspect of stimuli, whereas frontal neurons responded earlier to the top-down aspects. However, in their recordings, both the LIP and FEF contain both bottom-up and top-down signals at different times. Ibos *et al.* likewise recorded from the LIP and FEF simultaneously, finding that the LIP contained primarily bottom-up salience related signals. However, the LIP is not the source of the bottom-up salience signals [49], but rather inherits them from earlier cortex. In summary, like V4, the LIP biases bottom-up signals from the cortical route using top-down information from more frontal regions, although feature-specific modulation is observed less in the LIP. Based on this, we consider the LIP a feature-specific priority map (figure 3*a*).

#### (iv) Frontal eye field

The FEF is a region of the primate frontal cortex with robust eye-movement-related activity. Fernandes *et al.* [50] have recently recorded from FEF neurons while monkeys performed a visual search task in natural scenes, and they trained models to estimate spike rate, using either saccadic activity or salience model computation. There was little correlation between the saliency map and FEF activity in situations where the salient locations were not the eventual target of movement. In contrast, the FEF strongly responded to task-relevant, but non-salient stimuli, indicating that FEF activity implements a goal-related priority map rather than a bottom-up saliency map. Ogawa and Komatsu's recordings from the FEF in more artificial visual search tasks showed the same trend: FEF neurons' responses favoured the behavioural significance of the stimulus in their RF [45]. Results from Ibos *et al.* [49] likewise support this interpretation. Specifically, according to these authors, the FEF may be involved in endogenous attention (i.e. the representation of behaviourally relevant and goal-directed signals), although FEF neurons did also show some salience-like signals later than the LIP in the time course. This suggests that the FEF may receive bottom-up signals as input from elsewhere, for example via LIP. Finally, Thompson & Bichot [51] found that during a visual search task, FEF activity evolves during a fixation to represent non-feature-selective bottom-up information. However, the strongest firing neurons represent the region that would be the target of a saccade, even if the saccade is not executed. This is true even when there are stimuli that are more visually salient in the array, providing further support for FEF as a goal-related priority map (figure 3*a*).

### (b) Subcortical pathways

As described above, Yoshida *et al.* [39] have shown that attention guidance over complex natural scenes is preserved in the absence of V1. This directly challenges theories that crucially depend on V1 to compute low-level visual features guiding attention. Here, we review evidence that subcortical brain areas are involved in salience computation.

#### (i) Superficial layers of the superior colliculus

The SC is a phylogenetically old midbrain structure involved in visual control of orienting movements. In amphibians, reptiles, birds and lampreys it is known as the optic tectum, and it maintains much of the same function in mammals. Its superficial layers (SZ, SGS and fibre-rich layer SO) have strong visual responses, whereas the deeper layers (SGI, SAI, SGP and SAP) have activity related to orienting eye movements.

Anatomically, the sSC receives input primarily from the retina and visual cortex and sends outputs to the deeper layers in rodents [52] and primates [16,53–55], as well as relays input to other visually related structures including the thalamus. Physiological evidence that the superficial layers contribute to bottom-up salience has until recently been circumstantial: visual responses in SGS are stronger when the target is the focus of attention than not [56]. Furthermore, SGS neurons do not have strong tuning for any particular visual feature such as motion direction [57], colour [58] or orientation, although superficial layer neurons receive direct input from retina from the same population of retinal cells that send information to cortex [59]. Some SGS neurons respond invariantly to motion direction (pan-directional cells), but they respond more to moving than static stimuli [60]. This property is closely matched with the notion of feature-agnostic saliency map (figure 3*a*). Some directional selectivity has been seen in cats [61], rats [62] and mice [63], but our focus is on macaque monkeys, whose response characteristics are closer to humans. Recently, more direct evidence has emerged supporting salience signals in SGS. White *et al.* [38] recorded from SGS in primates during both free viewing and carefully controlled saccade tasks, and they found strong evidence that SGS activity is correlated with bottom-up salience of the visual input.

SGS is unique, because SGS neurons do not show feature tuning even though they receive feature-tuned input from V1 and other feature-tuned areas. This contrasts with other visual areas (such as V4) that receive similar feature-tuned input but *do* show feature tuning. Thus, unique feature-agnostic responses of SGS provide us further support for categorizing SGS as a saliency map analogue. On the other hand, it raises the question of how these feature-agnostic responses come about, and specifically what kind of information is transferred from V1 to sSC. Neurophysiological experiments combined with ablation or cooling of V1 have shown that the signal from V1 to SGS does not contribute to the RF properties of SGS neurons [21]. The same group also suggested that the V1 input may have a gating function in contributing to the control of the downflow of excitation from SGS to SGI [64]. These findings suggest that a feature-agnostic saliency map in sSC is less likely to be a product of V1 computation.

The lack of goal or eye-movement-related responses in SGS is also unique compared with other cortical areas, such as the FEF. Thanks to these unique patterns of connectivity and physiology, and its output to SGI [16,52,54,55,65], we look into more detail at the intrinsic connections of the SGS in §4, particularly to understand how a potential implementation of salience computation arises.

#### (ii) Deeper layers of the superior colliculus

Anatomically, SC deeper layers (dSC) receive converging associative inputs from cortex, basal ganglia and sSC [16,53–55,66]. Physiologically, SGI neurons are strongly related to (and can evoke) eye movements (overt attention). The SGI has also in recent years been the subject of more research related to covert attention. Fecteau and co-workers [2,67,68] have suggested that SGI activity is modulated by the locus of covert attention. Pharmacological inactivation of the intermediate and deep SC layers has been shown to negatively influence the ability of monkeys to perform attention-related tasks, but without having an effect on the enhanced response of neurons in the cortex (in this case, MT/MST) to attended locations [69,70]. Moreover, recording and inactivation experiments have demonstrated that these layers encode a real-time representation of behaviourally relevant goal location, independent of visual stimulation [4,71]. Finally, recent exciting results show that SGI neurons encode task- or goal-related priority even in the absence of bottom-up salience [37,38]. However, these responses are enhanced when the task-related target is also highly salient, suggesting that SGI receives and integrates information about both bottom-up and top-down conspicuity. Because the SGI then sends outputs directly to the brainstem oculomotor nuclei, this implies that SGI represents a priority map and is situated as the last stage of salience/priority pathways (figure 3*a*). At the circuit level, in contrast to the competitive nature of SGS, SGI acts as a stable integrator of its input [72,73], from which a winning target is selected via a combination of intrinsic and extrinsic computations whose nature is still under investigation.

#### (iii) Pulvinar

The primate pulvinar is a visual thalamic nucleus. Anatomically, the inferior section of the pulvinar (PI) receives input from sSC and has a retinotopic map. Physiologically, it is proposed to contain a representation of visual salience [74–76]. Pulvinar lesions in monkeys produce abnormal scanning of a complex visual array [77], providing evidence that the pulvinar is involved in salience computation during free viewing. Berman *et al.* [78] identified and characterized PI neurons receiving inputs from the sSC. The neurons' RFs had inhibitory surrounds, and direction selectivity was low [79]. This suggests that these neurons have similar characteristics with upstream sSC neurons and may inherit salience information from the sSC. On the other hand, PI neuron activity was not enhanced when the RF visual stimulus was the target of saccades. We classify PI into the category of feature-agnostic saliency map in figure 3*a*.

### (c) Differences in salience computation between cortical and subcortical pathways

In both cortical and subcortical areas, neurons process and represent successive stages of salience computation, starting with feature analysis and ending with bottom-up salience and top-down priority maps. We have described that some areas such as LIP and V4 can be classified into what we call feature-specific priority maps. In contrast, subcortical routes contain feature-agnostic representations in sSC and priority map-like representations in dSC.

We summarize our views on the neural correlates of salience computation in figure 3*b*. In terms of input stages, there is really no area in the brain for pure feature analysis, because even at the level of retinal ganglion cells, neuronal responses are influenced by surrounding visual input. In cortical pathways, information is processed from feature maps to feature-specific priority maps and ultimately to a priority map (black arrows in figure 3*b*). On the other hand, a subcortical route processes information in a feature-agnostic manner (through the grey lines in figure 3*b*). Although speculative, our hypothesis provides intriguing insights into how salience computation was evolutionarily built. The ‘bug detector’ neurons in the frog tectum [80] could be considered a phylogenetical ancestor of subcortical salience computation. Another speculation is that the cortical pathway may make it possible to use salience information for higher cognitive functions, such as covert attention, social gaze and working memory [14,81,82]. This distinction may be important functionally. The feature-agnostic saliency map in the subcortical route (with ‘bug detector’ neurons) may be optimized for salience computation, rather than detailed analysis of features. On the other hand, the feature-specific saliency map in the cortical route may be optimized for detailed analysis of features rather than for salience computation. The subcortical route can be useful for fast reaction, such as during free-viewing, whereas the cortical route can be useful for recurrent computation of bottom-up and top-down information, such as during conjunction visual search tasks [83].

## 4. Superior colliculus as a salience computer

We selected the neural pathways in §3 based on behavioural and physiological evidence demonstrating that each region might contain feature, saliency or priority maps. However, it is possible that these maps, in the computational sense, could be computed elsewhere in the brain and then inherited by other brain regions. For example, bottom-up signals in the FEF could be computed in the visual cortex and then inherited by the FEF. In order to understand what causes saliency-map-like activity, it will be necessary to understand the local implementation. This requires an understanding of the local computations of each region and the interactions between them. However, research into salience computation in the cortex has avoided delving into the particular implementation details of the local circuit. This is unavoidable—understanding local circuit dynamics in, say, the parietal and frontal cortex, while simultaneously accounting for their multitudinous inputs and outputs, is a daunting task. Nonetheless, exceptions do exist. For example, Li *et al.* [33] have detailed how spatial suppression mechanisms in V1 can lead to salience-like computations. Additionally, Soltani & Koch [84] constructed a spiking neural circuit model of salience computation in which cortical areas V1, V2 and V4 perform only lateral excitation/inhibition, and the final saliency map is represented in an identically implemented spiking neural sheet representing FEF/LIP. This type of full-scale model is important because it provides support for local computations to experimentally look for in each brain area. Although the Soltani model had shortcomings, such as small neural scale and physiologically unrealistic simplifications like synaptic weight decreasing with distance, it is the best existing neural model of the cortically implemented saliency map.

On the subcortical side, where local circuits are better understood, there has been more progress. The SC has a unique set of inputs and outputs that make it suited to salience computation and overt attentional control. Furthermore, as stated above, the SC has been shown to have saliency- and priority-map-like responses. For these reasons, the SC is the brain region currently most amenable to in-depth exploration at the circuit level.

### (a) Delving deeper: what is the superior colliculus doing in attentional control?

**Figure 4.**
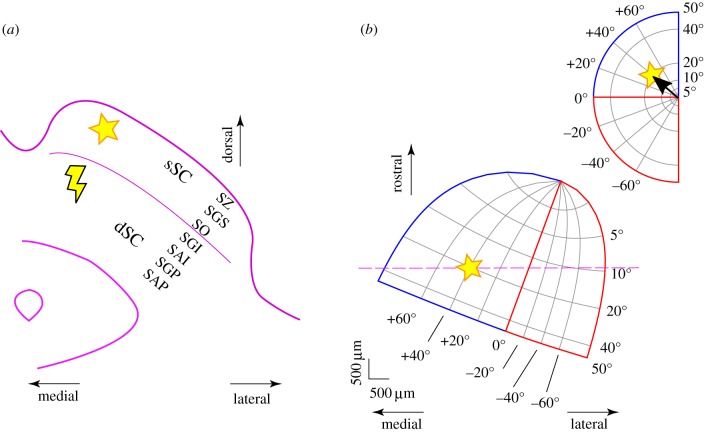
Major layers of the primate SC and their relation to visual and motor responses. The superficial SC and deeper SC are retinotopically organized and in spatial register. (*a*) Transverse slice through the brain showing the layers of the right hemisphere's superior colliculus. SZ, the stratum zonale; SGS, the stratum griseum superficiale; SO, the stratum opticum; SGI, the stratum griseum intermidiale; SAI, the stratum album intermediale; SGP, the stratum griseum profundum; SAP: the stratum album profundum. (*b*) The top-right inset shows visual field locations in polar coordinates. A stimulus in the visual field (star) evokes a topologically determined response in the sSC. A neural burst in the dSC directly ventral to the visual response (lightning bolt in *a*) will induce an eye movement which centres the star in the visual field (vector shown in the inset in *b*). The mapping between visual space and a horizontal slice through the SC is roughly log-polar [85], with a recently described areal bias towards the upper visual field [86].

Both sSC and dSC layers are organized retinotopically, and the layers are in spatial register with one another. A visual stimulus that evokes a neural response in the sSC can have the eyes guided to centre on that visual stimulus by a neuronal burst directly ventral to it, in the dSC (figure 4).

Behavioural and physiological evidence suggests rich intra-SC interactions that are critical for constraining computational models of saliency and priority map implementations. For example, results from tiny microsaccadic eye movements in an otherwise fixation-controlled cueing paradigm have shown that the local circuit in the SC may operate in a delicate balance, even during periods of forced fixation to a central stimulus [87–91]. In this regard, microsaccades are intriguing precisely because they reveal so much about the dynamics of the SC. During fixation, rostral SC activity has a strong influence on the selection of the next saccade target [90]. Thus, rostral SC activity must have an effect on any saliency or priority map present. Moreover, recent evidence from a variety of experiments is showing that microsaccades are part of the entire saccadic repertoire of the visual system, because they specifically and precisely realign the line of sight just like large saccades do [90,92,93]. Thus, even within foveal and parafoveal regions, the same issues of various objects competing for the line of sight also arise for microsaccades as they do for large saccades, and thus are equally as integral for understanding how the salience model could be implemented in the SC.

Results on microsaccades during peripheral cueing are additionally intriguing given the expansive spatial dissociation between the small microsaccade amplitudes and the peripheral stimuli [94]. Specifically, visual burst modulation (even in sSC) takes place if a stimulus appears in the far periphery near the time of a microsaccade [87]. Given that eye-movement-related bursts for microsaccades occur in the rostral SC region [91,95], where small eccentricities are encoded, this means that these bursts might interact laterally with more eccentric neurons in the SC map. Consistent with this, Ghitani *et al.* [96] have identified an excitatory connection from dSC to sSC that spans different eccentricities in sSC. This suggests that a saccade burst in one part of the map can be related to visual burst modulations in other parts of the map, implying that dSC may integrate part of the selection mechanism that outputs the location on the priority map to look at next.

Besides illuminating potential intra- and interlayer SC interactions, results from microsaccades also highlight additional constraints on saliency map computation. Namely, salience and priority are not stationary, static qualities of a scene or its internal representation. They are instead continuously modulated, whether by visual stimuli, or by generation of eye movements. Eye movements not only alter retinal images, thus remapping the retinotopically coded saliency and priority maps (figure 4*b*), but eye movements may also modulate intra-SC local activity patterns, thus altering either the saliency or the priority map [87]. An example of this is a scenario in which a visual stimulus suddenly appears while SC neurons are bursting to produce a microsaccade. In this situation, spatial read-out of the SC map will provide not a single saccade burst location, but instead multiple ‘hills’ of activation in the SC [94]. Thus, how the SC represents graded salience across multiple locations (i.e. as in a pre-selection graded saliency map) or a selected target (i.e. as in a post-selection priority map about to communicate the selected target downstream to eye movement centres) can dynamically change, and our understanding of the salience computation must account for this.

**Figure 5.**
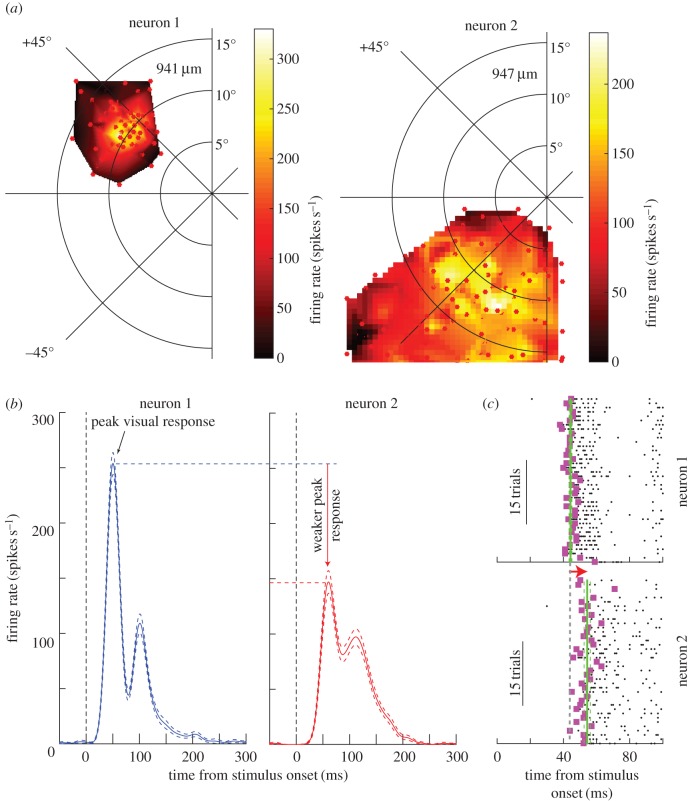
Functional asymmetries in the primate SC's spatial representation. (*a*) Two neurons from sSC in one monkey, matched for depth from SC surface and eccentricity, but in either the upper (neuron 1) or lower (neuron 2) visual field. The top visual field neuron has a significantly smaller RF size. (*b*) The visual responses of the same two neurons are different even when each neuron's preferred RF hotspot location is stimulated. (*c*) The latency to first visually induced spike is shorter in the upper visual field neuron. These results suggest putatively different patterns of lateral interactions in different portions of the SC map, which would be interesting to investigate from the perspective of what impacts such asymmetries have on saliency and priority map computation. Modified with permission from [86].

Alteration of the saliency and priority map representations in different retinotopic parts of the SC might also be expected in the light of recent discovery of strong functional and structural asymmetries in the primate SC [86]. Specifically, neurons in the upper visual field representation possess smaller RFs than neurons in the lower visual field representation (figure 5*a*), and this is true for both visual (sSC) and motor (dSC) RFs (figure 4*b*). Moreover, visual responses in the upper visual field are stronger than visual responses in the lower visual field, and they have shorter latencies (figure 5*b*). These results suggest that there is a functional discontinuity [86] in the retinotopic map of the SC. Importantly, the different RF sizes (figure 5) in different portions of the visual field are indicative of differing patterns of lateral interactions in different parts of the SC map. Similarly, voltage imaging of rat brain slices has suggested a rostral–caudal asymmetry in sSC, in which excitation preferentially spreads caudally within sSC. Intriguingly, this effect is strongest when the activity flows up to sSC from dSC [97]. These results have strong implications on the role of local SC circuit properties for attention, salience and priority control. For example, when multiple stimuli simultaneously appear, then one might predict differences in the trajectories of the evoked saccade (e.g. saccadic averaging [98]) depending on whether the stimuli were presented together either in the lower or upper visual field. Moreover, different population read-out schemes may exploit larger or smaller RF sizes in the SC's representations of the lower and upper visual fields, respectively, in order to serve attention. For example, illusory contour integration is perceptually better in the lower visual field [99]. If SC RFs act as a pointer to salient regions, then larger lower visual field RFs may aid in the integration that is necessary for disparate image regions associated with illusory contours. This being said, links between the SC asymmetries and attention need to be further investigated, especially given that spatial scales in other brain regions (such as V1) may not be asymmetric across the horizontal meridian like in the SC (and may even exhibit a mild asymmetry in the opposite direction).

Thus, converging evidence points to the SC as ripe for further investigation. We next highlight recent modelling work pointing to the feasibility of the SC as a structure capable of both performing feature-agnostic competition and integration with top-down information to select a target for overt attention. We argue that locally, the SC is algorithmically close to computational implementations of saliency maps. Although the majority of the work on the intrinsic SC circuit is based on rodent research, there is large preservation of the same circuitry in primates [100]. Slight differences in the numbers of horizontal cells, and the locations of retinal and cortical inputs, could have unpredictable effects on SC activity dynamics, but experiments with *in vitro* primate SC slices and modelling comparisons will be necessary to come to any concrete conclusions.

### (b) The local circuit of the superior colliculus

**Figure 6.**
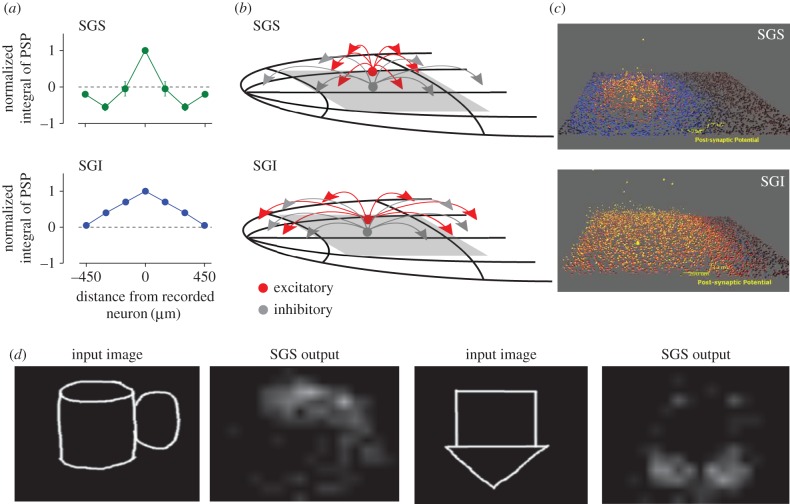
Simulation of internal computation in SGS and SGI. (*a*) Slice data from Phongphanphanee *et al.* [101] shows Mexican-hat in SGS and integration in SGI. (*b*) Best estimates of connectivity parameters (width of inhibitory and excitatory neuron axons and dendrites) using differential evolution Markov chain Monte Carlo by Veale *et al.* [102]. (*c*) Visualization of population activity in response to single pulse shows Mexican-hat in SGI, hill in SGI [102]. (*d*) Activity of slice model fit to SGS data in (*c*) in response to visual stimuli shows detection of salient positions. Two examples are displayed.

Phongphanphanee *et al.* [101] recently presented data from *in vitro* slice experiments of mouse SC showing that the intrinsic circuit of the superficial layers implements a centre-surround (‘Mexican-hat’) computation. In other words, lateral connections in the SGS cause competition between spatially adjacent stimuli (figure 6*a*, top). The extent of such lateral connections affects the spatial extent of the competition, and therefore has an impact on RF size of individual neurons. This, in turn, influences the size of the population of neurons that are simultaneously activated by a given stimulus (i.e. the ‘active population’). A recent study from rat SC also suggests interaction of competing activities within sSC [103]. At the level of the SGI, lateral interactions implement an integration mechanism, in which activity from nearby neurons is integrated proportional to their distance from one another (figure 6*a*, top). This means that the response of SGI neurons to various bottom-up and top-down locations is integrated in both space and time, evoking stronger activity and thus faster search times in cases where multiple bottom-up and top-down sources agree on the next target for attention. As in the SGS, the range of lateral interaction in the SGI has a bearing on the size of the active population for a given spatial location of a target. We thus hypothesize that the Mexican-hat computation in the SGS performs salience detection, and then the SGI integrates this with top-down goal information to select the next target for attention. Could the local circuit in the SC support these computations?

The sSC has been the subject of a fair amount of anatomical and physiological investigation. Historically, many cell types were identified based on morphology, but recent research has shown that there are four types of cells [52]. Most are excitatory: narrow-field vertical (NFV), wide-field vertical (WFV) and stellate cells (whether these are excitatory is a matter of debate [81]). In addition, one unequivocally inhibitory cell type has been identified: the horizontal cell. Horizontal cells have wide laterally spreading dendrites. In mice, only the NFV cells send projections to the dSC; other cell types send external projections mostly to the thalamus or to a sister nucleus known as the parabigeminal nucleus (PBg). The sSC receives excitatory inputs via axons from the retina and cortex.

There is less agreement on the classification of cells in the dSC [104,105]. As one moves deeper, there are increasing numbers of pyramidal cells, which project to the brainstem for evoking eye movements, but the most interesting region is the SGI, which contains a complex circuit of inhibitory cells and excitatory cells that exhibit the bursting properties associated with eye movement initiation. See [106] for details.

What causes the particular patterns of activity in isolated slices of the SGS versus the SGI? To better understand how these anatomical pieces combine to produce observed behaviour, Veale *et al.* [102] have recently applied advanced statistical methods to estimate the parameters of the SC local circuit that are most likely, given slice data from Phongphanphanee *et al*. Specifically, they applied a differential evolution/Markov chain Monte Carlo method to estimate the parameters of a spiking neural circuit model of the SC. Following the data from Phongphanphanee *et al.* [101], Veale and co-workers fit the SGS and SGI separately to reveal the most likely values of parameters such as lateral spread of inhibitory cells and excitatory cells, synaptic weights and synaptic parameters such as synaptic depression or facilitation. Examples of best parameter estimates, as well as visualizations of these simulations, are shown in figure 6*b*,*c*. These results work backwards from *in vitro* behaviour to support the hypothesis presented above: wide-reaching inhibitory cells and smaller excitatory cells in the SGS fit the slice data. The models use realistic densities of neurons and synapses based on anatomical findings, and in contrast to Soltani & Koch [84], modulate synaptic connection probability (rather than synaptic weight) as a function of distance. Using these models, Veale *et al.* [102] examined the computational properties of the SC simulations (Veale, R, Isa, T, Yoshida, M., 2015, Annual Meeting of the Society for Neuroscience). These authors specifically investigated how the circuit simulations respond to visual input (figure 6*d*). Although the spiking models were fit to physiological data of electrical stimulation from single electrodes, the firing of the output neurons in the superficial layers of the model shows a pattern in which areas of strong input are highlighted, and weak regions are suppressed. Based on these complementary physiological data and mathematical simulations, we conclude that the sSC can intrinsically compute a stable competitive filter of visual input, like the step of feature-agnostic saliency map (figure 2) of the Itti salience model [8]. The dSC could integrate this salience input with top-down information in order to transform it into overt attention shifts. However, to implement a winner-take-all mechanism in dSC, integration of topographically nearby inputs is not sufficient. Phongphanphanee *et al.* [101] argued that switching between linear integration and nonlinear burst generation is subject to control by the basal ganglia. Further studies are needed to clarify how winner-take-all computation is implemented by circuits within dSC or downstream areas.

One question that remains is whether the SGS is sufficient to compute the saliency map or whether the more superficial SZ layer or the fibre-rich deeper layer SO might also play an important role. It is already known that SO contains a large population of a different cell type, the wide-field vertical (WFV) cell [107], which is not implemented in Veale *et al*.'s model. Because WFV cells are excitatory neurons with large spreading dendrites, it is possible that they may play a role in salience computation by integrating information over a larger region than the neurons in SGS with narrower dendrites. Incorporating WFV cells into Veale *et al*.'s model may contribute to reconstructing how sSC signals are transferred into the dSC *in vivo*.

Another intriguing possibility comes from Vokoun *et al.* [103], who imaged parasagittal slices of rat SC. Stimulating two locations in the SGI causes a strong flow of neural activation back to the SGS, which spreads laterally and interacts based on the distance between the stimulated points. The interaction results in either two separate peaks of activation when the stimulations are distant, or a single-averaged peak in the middle when the stimulations are adjacent. This phenomenon could work to pipe the goal-directed and multisensory priority information present in the dSC back up to the competing saliency map representation in the sSC. From the sSC, the combined top-down and bottom-up information could then be re-integrated with higher-level areas via the thalamus or directly evoke overt attention shifts via the sSC–dSC pathway. However, White *et al.* [37,38] did not report priority responses in sSC cells. Thus, the dSC–sSC pathway needs to be examined in more detail to understand whether it functions differently between primate versus rodent.

Finally, it is important to connect salience computation in the SC back to overt attention. In other words, how is the next target for fixation selected by the graded saliency and priority maps? We can use information from the saccadic averaging literature to constrain the possible shapes of the neural circuits that perform this final target selection. The strongest constraint is that any circuit we propose to perform target selection must produce saccades that obey the linear vector averaging of multiple saccade targets in visual space. The proposed selection circuit must perform this correction despite two nonlinear transformations: from linear visual space to the log-polar neural representation, and then back to linear vector space for the eye movement [98]. It is also possible that such a correction from nonlinear to linear summation is likely computed downstream of the SC (e.g. in cerebellum). One intriguing possibility is that recurrent circuitry in the SC could perform these nonlinear-to-linear corrections [108]. Further studies including both experimental and theoretical methods will be needed to clarify this question.

## 5. Conclusion

In this review, we overviewed how salience computation is implemented in attention-related visual pathways. Based on findings in neurophysiology and functional imaging, we classified the responses of each brain area as being computationally equivalent to one of the stages of salience computation (figure 3*a*). We emphasized a possibility that there are two partly segregated pathways for salience computation, namely the cortical and subcortical pathways. Finally, we zoomed in to focus on the subcortical pathway to examine in greater detail the algorithmic equivalence of the brain implementation of the salience model. The SC represents a visual saliency map via centre-surround inhibition and winner-take-all mechanisms built into the local circuit, thereby affecting saccadic and microsaccadic eye movements. Lateral interactions in the local SC circuit are particularly important for controlling active populations of neurons. This, in turn, may help explain long-range effects, such as those of peripheral cues on tiny microsaccades. Finally, we showed how combination of *in vitro* neurophysiology and a large-scale computational modelling is able to clarify how salience computation is implemented in local circuits of the SC.

Several open questions remain to be investigated. How are the Mexican-hat function in the sSC and the integration and winner-take-all function in the dSC evolutionarily and developmentally built? What are the computational benefits of such structures? More specifically, we would like to know whether such structures in the sSC and dSC are optimized for covert attention and eye movements, respectively. What is the fundamental difference between cortical and subcortical salience computation? Finally, the functional asymmetry in SC (figure 5) suggests that future studies will reveal critical differences in structural and functional architecture that characterize subcortical and cortical salience computation.
